# Conceptualisation, estimation, and empirical analyses of land–sea convergenomics: A case study on Bohai Economic Rim cities

**DOI:** 10.1371/journal.pone.0274707

**Published:** 2022-09-20

**Authors:** Zhe Yu, Qianbin Di

**Affiliations:** 1 School of Geography, Liaoning Normal University, Dalian, China; 2 Marine Economies and Sustainable Development Research Center, Liaoning Normal University, Dalian, China; Northeastern University (Shenyang China), CHINA

## Abstract

This study proposes a land–sea convergenomics model based on analyses of marine and terrestrial economies. A viscosity-based system of indices was constructed to evaluate the land–sea convergenomics of Bohai Economic Rim (BER) cities and was applied to analyse the viscosities of BER cities from 2009 to 2019. BER cities’ viscosities trended upward, although with significant disparities. The developmental fundamentals were high at the system level and grew steadily during the study period. Resource development was low with a slow growth rate. Industrial linkages grew significantly but exhibited minor fluctuations. Outcome creation began at a low level but grew steadily. Cluster analyses revealed that, overall, the development of land–sea convergenomics in the BER occurred in clusters, with viscosity distribution centred around three core cities: Tianjin, Qingdao, and Dalian. Due to regional disparities, the effects of labour productivity, science and technology investment, economic fundamentals, and energy efficiency on land–sea convergenomics were significantly differentiated.

## Introduction

Marine and terrestrial domains are inextricably bound by their mutual reliance and influence. As China possesses both marine and terrestrial domains, implementation of coordinated strategies that simultaneously develop both economies would prove beneficial. However, the traditional ‘land-dominated’ approach to development has hindered this process. Furthermore, the advancement of marine development has caused many conflicts with land development, while the ensuing competition for space and resources has exacerbated the divide between said domains’ economies. Therefore, a new model of high-quality development in which marine and terrestrial economies are integrated via ‘sea-driven’ and ‘land-supported’ approaches, is needed [1–4].

At present, China is in a critical period of strategic adjustment of its economic structure and rapid development of the marine economy. Therefore, researching a new type of marine and land integration economy that adapts to the contemporary development situation is necessary to promote high-quality, coordinated, and sustainable development of the regional marine and land economy. Through the empirical measurement of the land–sea convergenomics in the Bohai Economic Rim (BER), the characteristics and laws of this mode are presented to guide the coordinated and high-quality development of the sea and land economy.

The ‘coordinated development of marine and terrestrial economies’ is a concept that has emerged from making ‘coordination’ the primary focus of efforts to manage the relationship between marine and land economies. ‘Land–sea coordination’ was first proposed by Chinese researchers [5, 6]; studies in this area have focused primarily on the effects of marine economies on the economic development of coastal regions and quantification of the relationship between marine and terrestrial economies [7–10]. Contrarily, researchers outside China have focused on the integrated management of coastal and marine regions and the role of the marine economy in the development of local or national economies [11–15]. The convergence of marine and terrestrial economies, that is, convergenomics, is inevitable and may be discussed in two aspects. The first pertains to externalities, such as accepting this ideology in mainstream development and realising the requisite scientific and technological progress and implementation. The second pertains to internal drivers, such as the fundamental relationships between marine and terrestrial industries. Indeed, convergenomics is the future of economic development in China, as it is critical for the expansion, optimisation, and competitiveness of industries [16].

To date, research on marine and terrestrial economies has focused primarily on analysing the correlation and coordination of metrics or industrial indices related to land–sea interactions, unification of marine and terrestrial economies, and coordination of marine and terrestrial development [17–22]. However, few studies have reported on the fundamental mechanisms and modes of land–sea convergenomics. Furthermore, no multidisciplinary or multimodal empirical studies exist on cross-regional land–sea convergenomics at the city level and its factors based on the outputs of marine and terrestrial economies. The research on the sea–land integration economy is scattered and one-sided, lacking a certain degree of systemisation. The currently available research mainly focuses on the qualitative analysis of the realisation path of sea–land integration and the integration development of a certain aspect of simplification, as well as pure technical integration research [23–26]. Few sources fully consider the mutual influence and interaction between the marine economic system and the land economic system. There is also a lack of quantitative analysis of the land–sea convergenomics.

Herein, we promote land–sea convergenomics that aligns with the current linkage development of the marine economy and land economy and provides theoretical support for the evolution of the entire enormous economic system to a higher level. We incorporate the concept of viscosity into a systems theory framework to conduct detailed analyses of the economic mechanisms and modes of land–sea convergenomics based on previous research findings, culminating in the construction of a viscosity-based system of indices for the evaluation of land–sea convergenomics in Bohai Economic Rim (BER) cities [27, 28].

**Fig 1:**
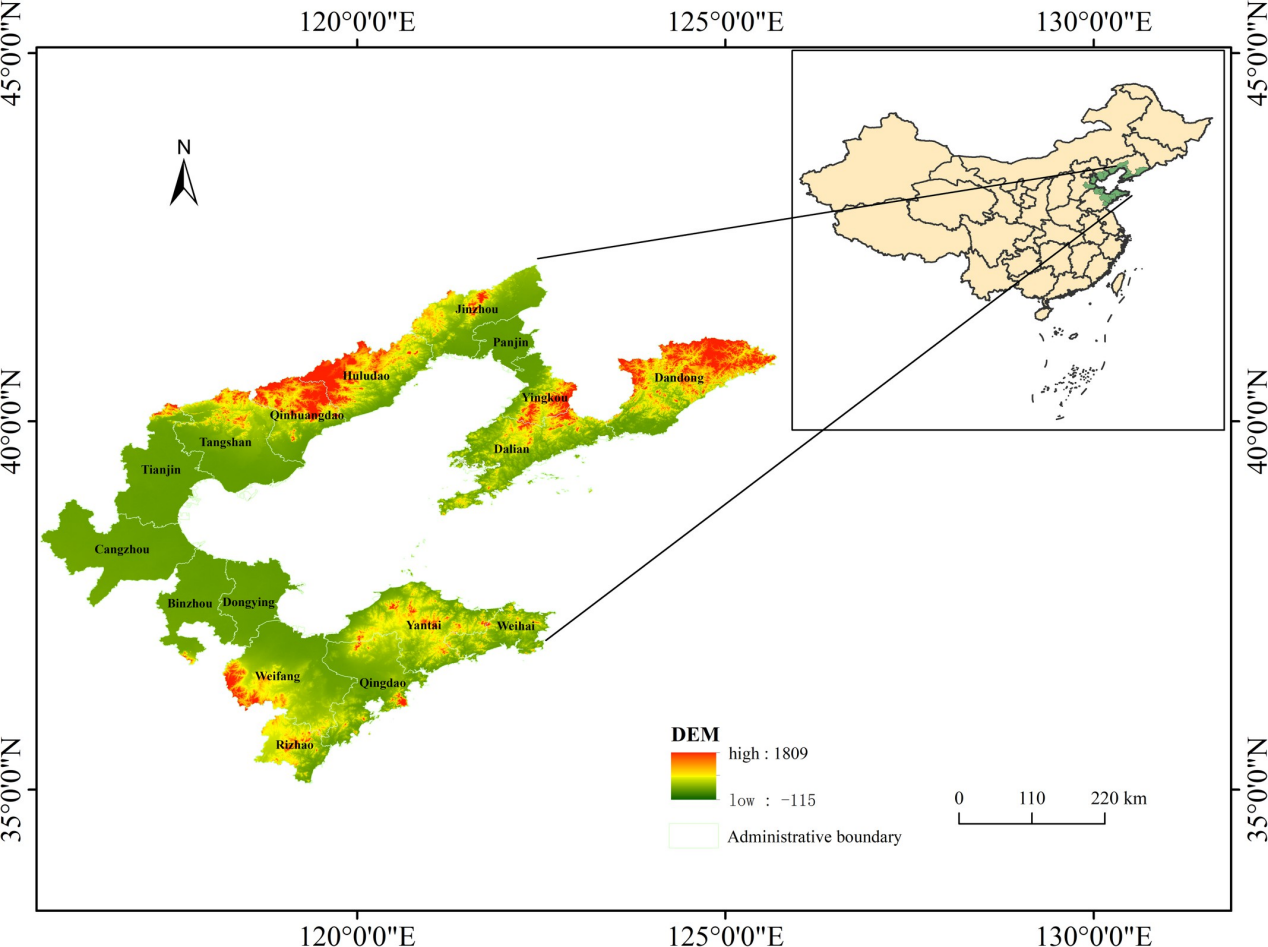
Location of the study area and the Circum-Bohai Sea, China.

The Bohai Sea region is one of the three major maritime economic regions in China (Fig 1). Owing to its advantageous geographical location, rich and diversified resources, and progressive industry and technology, it has become the backbone of China’s maritime economic development. In the context of accelerating high-quality development of China’s marine economy, the evaluation of land–sea convergenomics in the Bohai Economic Rim (BER) has important theoretical value and practical significance. The evaluation clarifies the current situation of land–sea economic development in coastal cities and assists in the formulation of measures that conform to the different characteristics of various regions.

On the research scale, the cross-regional analysis at the city level, which considers regional particularity and regional relevance, is beneficial to—and will play a practical role in—regional planning. Owing to the context of this study, its scale and sample limitations, we focused on the city level and selected 17 BER cities for analysis. For empirical measurement and method selection and application, we adopted multi-disciplinary and multi-domain methods. Optimal entropy and polygonal methods are used to quantitatively evaluate the land–sea convergenomics viscosity values of these cities from 2009 to 2019 and to investigate their spatiotemporal characteristics and dynamics. Spatiotemporal hotspot detection (using SaTScan v9.5 software) and the geographical and temporal weighted regression (GTWR) model are used to perform spatiotemporal clustering and factor analysis on land–sea convergenomics. This study elucidates the internal mechanisms, evolutionary patterns, and factors of land–sea convergenomics in the BER and contributes to the development of marine and terrestrial economies in this new era in China.

### Concept analysis, measurement of viscosity, and research methodology

#### Concept analysis

In 1978, Yu Guangyuan, a famous economist, proposed the concept of “marine economy”, and the theoretical research on China’s marine economic system has continuously developed and improved thereafter. Since the 1990s, with the deepening of development activities, diversified perspectives and marine economic research methods continue to emerge, including the upgrades and development of concepts such as land–sea coordination and land–sea integration. Simultaneously, theoretical research has expanded from the work of a single marine economics department to a comprehensive marine economics field of study and from studying marine resources to research on the impact of marine industry development on the national economy, ecology, and social evolution.

Land–sea convergenomics is a sea–land economic integration mode or approach to the perspective of land–sea economic coordination and integration under the new concept. The characteristics and laws of the convergenomic mode are determined through empirical measurement to guide the coordinated development and high-quality development of the land–sea economy. Therefore, land–sea convergenomics is a more deeply coordinated development of the land-and-sea economy.

In systems theory, a system is an entity consisting of mutually interconnected and constraining elements. Furthermore, land–sea economies are complex systems consisting of many subsystems and elements [29–31]. Industrial integration is an innovation-driven phenomenon that can only exist within an open system and manifests as inter-industrial competition and cooperation, the co-evolution of multiple factors and the extension of other processes that contribute to industrial integration. Marine and terrestrial economies are deeply interwoven due to their mutually beneficial relationship, which could become even stronger because of technological progress and coordinated land–sea development strategies [32–35].

This study proposed ‘land–sea convergenomics’, a systems theory framework that views marine and terrestrial economics through the lens of industrial integration. It is consistent with the processes of marine and terrestrial economics and has absorbed lessons from previous conceptual frameworks, such as ‘land–sea coordination’ and ‘the unification of land and marine economies’, while remaining distinct [36–39]. Studies on land–sea coordination mainly focus on the ideological connotations of their respective frameworks; the planning of land and sea economies ensures their development in a coordinated and harmonious manner. The concept of unifying the land and marine economies eliminates common barriers in the development of marine and land industries and facilitates their co-development. From the perspectives of economic integration, socialised mass production, and the internationalisation of labour division, national economies are driven to cooperate and integrate. Land–sea convergenomics is guided by the ideas of land–sea coordination and is a framework for analysing industrial interactions, resource complementation, geographic interconnections, and feedback mechanisms in the co-development of marine and terrestrial industries. Indeed, the convergence of marine and terrestrial economies could contribute to China’s aims to reduce carbon emissions and achieve carbon neutrality. This convergence process will involve changes in economic structure, as the elements in the system will be reorganised to find their optimal configuration, which will enhance factor productivity, create new modes of progress, establish new industrial connections, and diversify business models, thus enhancing economic vitality (S1 Fig). The systems of land–sea convergenomics include factors such as economic fundamentals, resource development and utilisation, technological innovation, and socio-economic environment, which are all related by non-linear relationships.

### Definition and measurement of land–sea convergenomics viscosity

From a relational perspective, parallels exist between fluid physics and land–sea convergenomics, as the latter is a continuously evolving and open system that consists of many interconnected and co-evolving factors [40]. In convergenomics, the connections and constraints inside the system are akin to a form of friction that causes the system to organise itself into an ordered state. We refer to this intrinsic attribute as the ‘viscosity’ of land–sea convergenomics, which pertains to the interconnections and interactions between the internal system factors in land–sea convergenomics [41–44]. Although land–sea convergenomics differs from previous economic frameworks, the principles revealed by viscosity theory apply to convergenomics phenomena. Therefore, we incorporated the concept of viscosity into this framework.

Guided by system theory, the system is a set or whole comprising many interrelated elements and restricted parts. Studying the problem from the perspective of the system is useful for defining and grasping the entirety of the problem, drawing on the previous research contents of land–sea coordination and sea–land economic integration [45, 46]. Based on the internal characteristics of the land–sea economy and the development process and using the land–sea convergenomics system as a guide, we divided the land–sea convergenomics system into layers. In an open and integrated system, non-linear motions between the system factors induce a form of internal bonding between the factors, that is, land–sea convergenomics viscosity [47]. Based on a review of the relevant literature, a systems theory framework was used to analyse the following system aspects, convergenomic development fundamentals, convergenomic resource development, convergenomic industrial linkage and convergenomic economic outcomes. The selection of each index in the system layers reflects the purpose of the joint action of cities and oceans and introduces the index content in detail to explain the effectiveness and scientificity of the evaluation index.

Convergenomic development fundamentals refer to the foundations of convergenomic development, as they affect the operational structure and scale of development. Herein, the level of economic development and position of land/sea industries in local economic development was evaluated using the gross products of the land and sea economies, their percentage within the GDP, and the location quotients of the marine and terrestrial industries. Convergenomic resource development is an important aspect of economic development in land–sea convergenomics, as it pertains to converting resource abundance and control over the land–sea environment into resources that can contribute to economic development. Therefore, it requires abundant resources, an amicable ecological environment, and advanced technologies. Herein, the abundance of land–sea resources was assessed using the resource abundance metric, while the state of the land–sea environment was evaluated based on land–sea environmental pressure and capacity. Specifically, the abundance of sea and land resources was measured by green space area, crop sowing area, output of marine aquatic products, and mariculture area. The state of marine and land ecological environments was expressed by the pressure and carrying capacity of the marine and land environments, in particular, the proportion of the four inferior seawater types, amount of industrial solid waste, pressure level, and environmental quality and carrying capacity of seawater. Environmental quality includes the comprehensive utilisation rate of industrial solid waste, harmless treatment rate of domestic waste, and centralised treatment rate of sewage treatment plants. By using sea land scientific and technological innovation, as well as scientific research and technology to serve geological practitioners, the level of resource development can be expressed. Energy consumption value per 10,000-yuan GDP and GDP growth rate reflect development capability and power [48, 49].

Advanced technologies and resource development capacity are necessary to establish an integrated land–sea economy; convergenomic industrial linkages are central to land–sea convergenomics; and the integration of resources, efficient logistics, and industrial linkages help increase the flow of finished goods to urban centres and strengthen inter-industrial interactions, thereby promoting the co-development of land and sea economies. This study evaluated the logistics level based on port cargo throughput, road freight volume, and traffic accessibility (per capita road area). The linkage of tertiary land and sea industries, derived from the ratio of marine to land industry changes, was used to assess industrial interactivity. Convergenomic outcome creation is a fundamental requirement of land–sea convergenomics, as it influences economic development to benefit society. Furthermore, labour and investment are important for developing converged land and sea economies; therefore, labour participation was assessed using the number of registered unemployed persons in the city; investment, using the amount of fixed asset investment in land and sea industries; and social development, using the rate of urbanisation and quality of life.

By incorporating the concept of viscosity into land–sea convergenomics, a comprehensive system of 23 indices was constructed to adequately reflect each aspect of converging land and marine economies. The selection criteria for the indices included scientific reproducibility, mutual independence, and measurability (Table 1).

**Table 1:** Evaluation system for land–sea integration and economic development cities of Circum-Bohai Sea.

Target layer	Systems layer	Index layer
	Convergenomic development fundamentals	Gross marine product
		Percentage of GDP of the gross marine product
		Gross terrestrial product
		GDP percentage of the gross terrestrial product
		Location quotient of marine industries
		Location quotient of terrestrial industries
	Convergenomic resource development	Resource abundance
		Environmental load of the land–sea environment
Viscosity of land–sea convergenomics in BER cities		Environmental carrying capacity of the land–sea environment
		Level of development
		Capacity for development
		Drive for development
	Convergenomic industrial linkages	Port cargo throughput
		Road freight volume
		Traffic accessibility
		Linkage of primary marine and terrestrial industries
		Linkage of secondary marine and terrestrial industries
		Linkage of tertiary marine and terrestrial industries
	Convergenomic outcome creation	Rate of urbanisation
		Number of registered unemployed persons in the city
		Fixed asset investment in marine industries
		Fixed asset investment in terrestrial industries
		Quality of life

### Research methodology

#### Land–sea convergenomics viscosity assessment model

The entropy method is an objective-weighted method in which the weight of an index is determined by its entropy. Compared with the subjective assignment method, the entropy method offers stronger objectivity, higher accuracy, and broader adaptability [50].

The ordered polygon area method takes a fixed point as the common point of multiple line segments and extends outward to form a polygon. Each common point represents a specific variable, while the length of each line segment reflects the corresponding index value. The area of each triangle formed by the adjacent line segments of the common point is calculated via ordered arrangement to obtain the polygon area, which is then used as the comprehensive index value. O is the origin, while OI, OC, OE, and OU represent the basis of integrated economic development, integrated economic resource development, integrated economic and industrial linkage, and integrated economic achievements, respectively; the area of the quadrilateral is defined as the economic viscosity value of sea–land integration [51]. Considering that the elements of the sea land fusion system overlap, the entropy method and ordered polygon area method effectively assess the economic viscosity of sea land fusion.


$$\eta =\frac{1}{2}\sin\alpha \left(OI\times OC+OC\times OE+OE\times OU+OU\times OI\right)$$ (1)

$\alpha =\frac{360{}^{\circ}}{4}$ *η*: Level value of the economy of marine and terrestrial integration.

#### Spatiotemporal agglomeration

Space-time permutation scan statistics is a clustering method used to detect whether aggregation and random distribution significantly increase in the spatiotemporal range. In recent years, spatiotemporal scanning statistics has been applied in the fields of geography, sociology, and economics and has achieved good results. Kang [52], as well as Maoh and Kanaroglou [53] have extended it to the field of social economy. This study used retrospective spatiotemporal rearrangement scanning statistics and SaTScanV9.5 software to analyse the temporal and spatial agglomeration of sea–land integration economic viscosity of cities around the Bohai Sea. Kulldorff [54] proposed a retrospective spatiotemporal scanning statistical method, as well as a spatiotemporal rearrangement scanning statistical method [55]. Meanwhile, the cylindrical scanning window scans and counts different spatiotemporal regions. As such, spatiotemporal aggregation regions are explored from different spatiotemporal radii and analysed using the Monte Carlo method.

#### Geographical and temporal weighted regression model

The GTWR model accounts for spatial and temporal non-stationarity and closely reflects true economic activities. Huang et al. [56] proposed a GTWR model considering temporal and spatial non-stationarity as an extension of the traditional geographically-weighted regression model [57, 58]. This model can incorporate the spatiotemporal characteristics of data into the regression model through a series of panel data processing to effectively reduce the model error and parameter estimation error. The formula is:

$$y_{i}=\beta_{0}(u_{i},v_{i},t_{i})+\sum_{k=1}^{m}\beta_{k}(u_{i},v_{i},t_{i})x_{ik}+\varepsilon_{i},i=1,2,\ldots \ldots ,n$$ (2)

where *y*_*i*_ is the dependent variable of the *i* sample point, *x*_*ik*_ is the value of the *k* independent variable at the *i* sample point and (*u*_*i*_,*v*_*i*_,*t*_*i*_) are the spatiotemporal coordinates of the *i* sample point. *β*_*k*_(*u*_*i*_,*v*_*i*_,*t*_*i*_) is the regression coefficient of the *k* independent variable at the *i* sample point; *β*_0_(*u*_*i*_,*v*_*i*_,*t*_*i*_), spatiotemporal intercept of the *i* sample point; *ε*_*i*_, residual.

### Data sources and normalisation

The data sources used in this work include the 2010–2020 editions of the China Marine Statistical Yearbook [59], China City Statistical Yearbook [60], Shandong Statistical Yearbook [61], Liaoning Statistical Yearbook [62], Hebei Economic Yearbook [63], Tianjin Statistical Yearbook [64], Bulletin on Environmental Quality of China’s Coastal Waters [65] and the data reported in the statistical bulletins of the relevant national departments.

The extreme difference method was used to normalise raw data and eliminate the effects of dimensional differences.

## Empirical analysis of land–sea convergenomics viscosity in BER cities

### Changes in land–sea convergenomics viscosity

**Fig 2:**
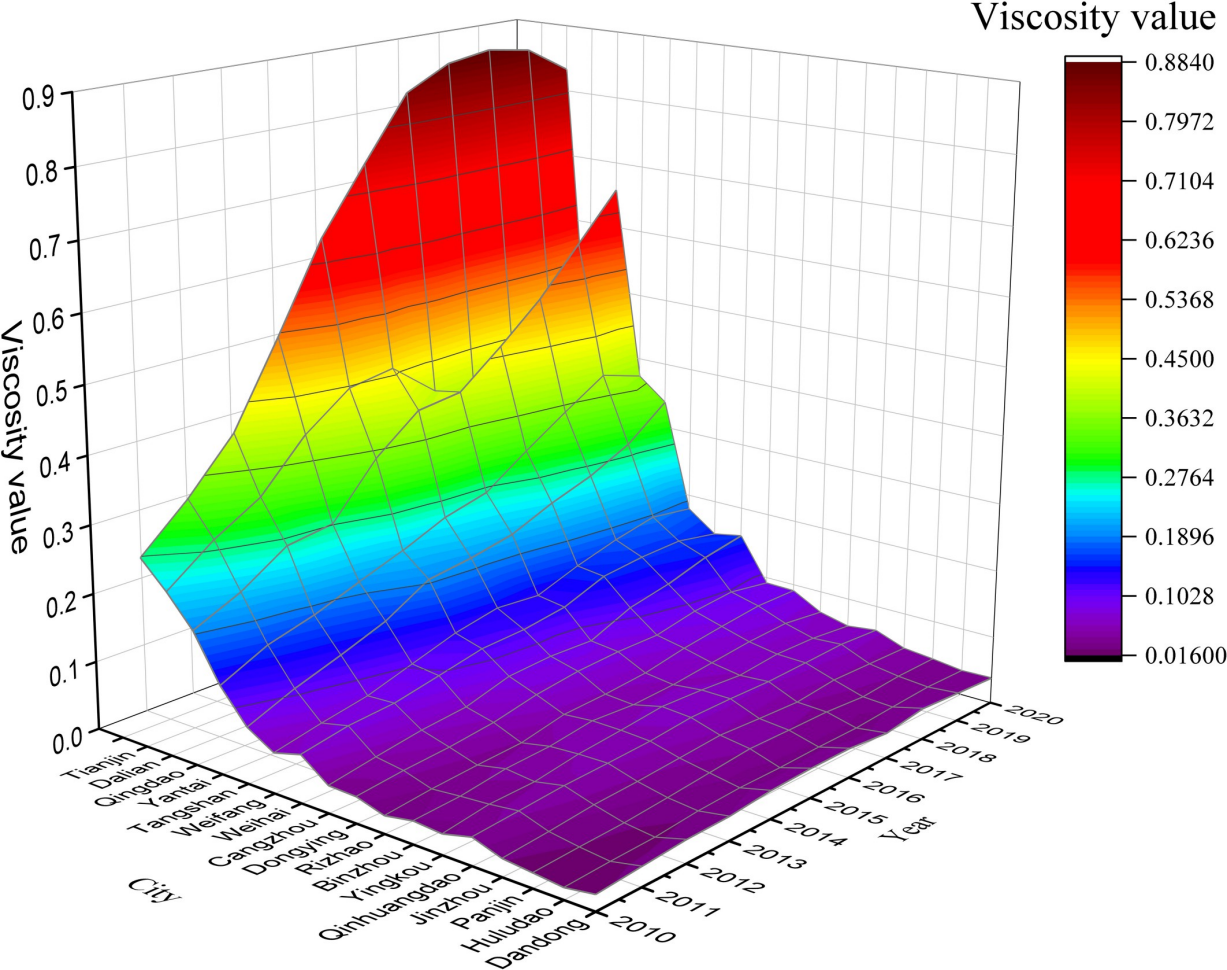
Level of the land–sea convergenomics in cities of Circum-Bohai Sea (2009–2019).

Fig 2 and S2 Fig show that the viscosities of BER cities trended upward; however, there were large differences among them. Viscosity ranged from 1.333 to 3.613, which increased from 2009 to 2018 and exhibited a slight drop in 2019. Each city had a different starting point and rate of increase. Some cities showed large increases; however, most exhibited small, fluctuating increases and low viscosity (0.017–0.186). Tianjin, Qingdao, Dalian, Yantai, and Tangshan had high starting points and showed large increases in viscosity. Tianjin had the highest viscosity among the BER cities at all time points, increasing rapidly to 0.883 after 2011 and dropping slightly after 2017. Those of Qingdao and Dalian initially trended upward; however, Dalian had large fluctuations after 2014. Meanwhile, after 2015, the viscosity of Qingdao (0.658) was greater than that of Dalian. Although the viscosity of Dalian exhibited small increases in the subsequent period, the difference between Dalian and Qingdao ultimately became quite large.

At the systems level, the convergenomic development fundamentals were high in all BER cities and improved steadily from 2009 to 2019. The convergenomic resource development was low throughout the study period, with a slow growth rate. Convergenomic industrial linkage showed the largest growth and highest scores of all systems, with small fluctuations. Although the convergenomic outcome creation started low, it maintained a high and steady growth rate. The cities with high starting viscosities and large increases in viscosity also showed large system-level percentages. Tianjin, Qingdao, Dalian, and Yantai showed very high percentages in all systems. Tangshan had lower resource development and outcome creation percentages than the other three cities but showed a high percentage of industrial linkages. Its outcome creation percentage also increased continuously, indicating that it made good use of its industrial linkage advantages.

These findings indicated that the BER had not reached its optimal state in terms of land–sea convergenomics. The city-to-city disparities were large, with few large cities exhibiting rapid increases in viscosity and most showing low viscosity and growth rate, indicating that the development of land–sea convergenomics was highly reliant on pre-existing economic resources and logistical infrastructure with a lack of new business models and innovation. Most cities showed low and slow-growing resource developmental scores, indicating room for improvement in technological progress and ecological environment.

### Spatiotemporal distribution of viscosities

**Fig 3:**
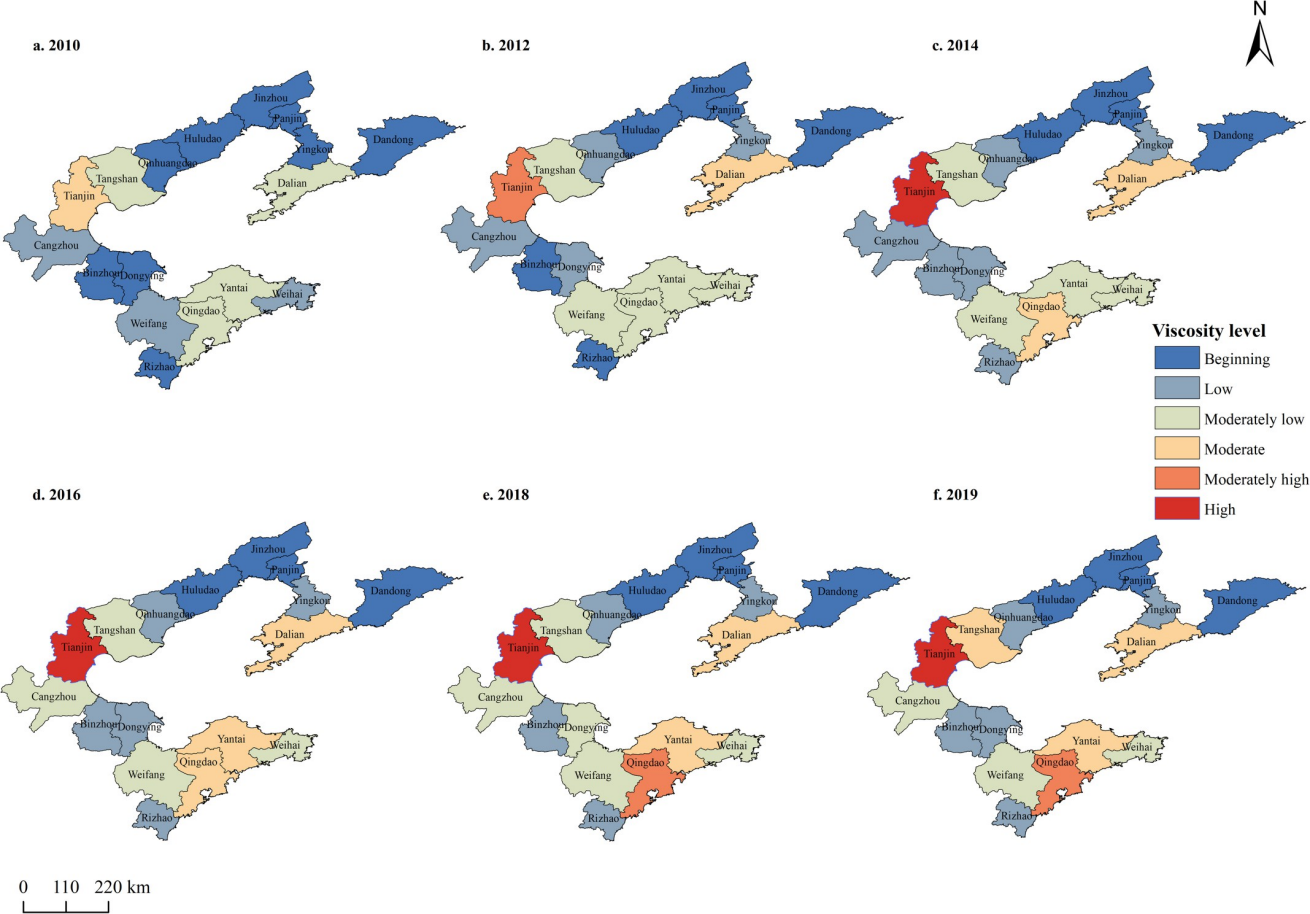
Grade change distribution of the land–sea convergenomics level in Circum-Bohai Sea cities (2009–2019) (a-f).

To elucidate the spatiotemporal pattern of viscosity development, the 2009–2019 viscosities of the BER cities were categorised into six levels: high, moderately high, moderate, moderately low, low, and beginning (S1 Table). Changes in viscosity were found to be spatiotemporally differentiated across the BER cities (Fig 3).

Fig 3 also indicates that the viscosity distribution was centred around the three core cities (Tianjin, Qingdao, and Dalian). The central region reached its highest level in 2014, with Tianjin showing the fastest development. Cities such as Tangshan and Cangzhou, which lie in the sphere of influence of Tianjin, improved to medium and moderately low levels. Qingdao quickly improved from a moderate level to a moderately high level in the southern region, while cities in its vicinity, such as Yantai and Weifang, also improved to moderate and moderately low levels. Though far from the central cities, Qinhuangdao, Binzhou, Dongying, Rizhao, and Weihai were affected by their corresponding central cities; however, their viscosities only improved slightly because of their lack of developmental fundamentals and distance from the central cities. Dalian, the central city of the central region, developed from a moderately low to a moderate level, where it stagnated. Consequently, its influence on the surrounding cities was small. Overall, the development of land–sea convergenomics in BER cities was centred around Tianjin, Qingdao, and Dalian. However, Dalian had a less pronounced influence on its surrounding cities, compared with Tianjin and Qingdao; its development in this respect also stagnated because of various constraints.

### Spatiotemporal cluster analysis of land–sea convergenomics

According to the characteristics of the sea–land integration economic data of cities around the Bohai Sea, retrospective spatiotemporal rearrangement scanning statistical analysis was employed to investigate spatiotemporal aggregation. To facilitate software calculation, this study expanded the economic viscosity of sea–land integration for cities around the Bohai Sea from 2009 to 2019 by 10,000 times and applied the current spatiotemporal hot spot scanning technology SaTScan V9.5 [66]. Fifty percent of the total research period was set as the time scale with one year as the unit. The results were analysed statistically via Monte Carlo simulation with a significance level of p < 0.0001; the results were visually expressed using ArcGIS10.2 software (S3 Table).

**Fig 4:**
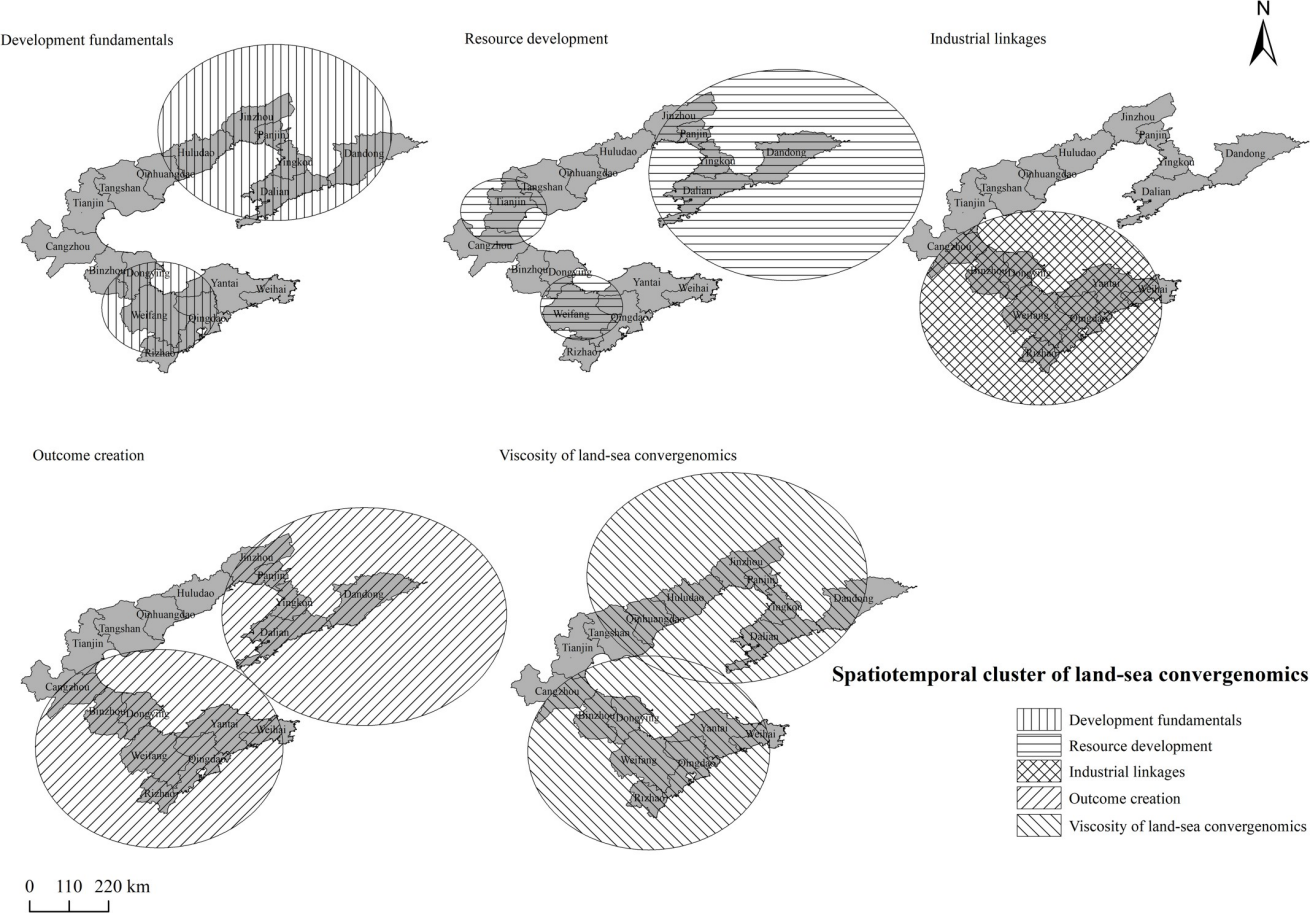
Spatial-temporal clustering map of the land–sea convergenomics in Circum-Bohai Sea cities.

Based on system-level spatiotemporal cluster analysis (Fig 4), the development fundamentals were clustered in two blocks, around Qingdao-Weifang and Dandong-Huludao. Resource development was clustered in three regions, around Panjin-Dandong, Tianjin, and Qingdao-Weifang. Industrial linkages were clustered in one region—the coastal cities of Shandong province. Outcome creation was clustered around two regions—the coastal cities of Shandong province and Jinzhou to Dandong. The clusters coincided with the development fundamentals clusters; however, they were wider. The viscosity clusters overlapped with those of the four previously mentioned systems, among which outcome creation showed the widest cluster spread, indicating that the contribution of land–sea convergenomics to social development was a priority in all cities. The clustering of developmental fundamentals and resource development largely overlapped, except in Tianjin, where the resource development cluster was smaller, indicating that the land and sea resource development capacity in Tianjin was higher than that in other cities. Industrial linkage clustered around the coastal cities of Shandong, indicating that these cities had conditions that were highly applicable to the industrial linkage between terrestrial and marine industries. Each previously mentioned system was distinct, yet they exhibited some overlap within their clusters. Hence, land–sea convergenomics developed in a clustered manner among the BER cities. Among the four systems, outcome creation showed the greatest improvement. Developmental fundamentals and resource development showed approximately equal progress. In contrast, industrial linkage was largely concentrated in the coastal cities of Shandong, indicating that additional efforts should be made to develop land–sea convergenomics and its systems in a coordinated manner in that area.

## Spatiotemporal analysis of BER land–sea convergenomics factors

We investigated the spatiotemporal distribution of viscosity in the BER using the GTWR model, which provides a spatiotemporal perspective of the viscosity differentiations caused by each factor.

Stepwise regression was performed using SPSS software to determine the optimal combination of independent variables [67–69]. The variables were labour productivity (GDP/worker), economic fundamentals (GDP), science and technology investment (expenditure on science and technology), and energy efficiency (consumption per ten thousand CNY). The previously computed viscosities were used as the dependent variable. Multicollinearity tests were performed on the independent variables by calculating their tolerance and variance inflation factors, which were generally > 1 and < 10, respectively. Therefore, there was no multicollinearity between the independent variables. Finally, the ordinary least squares regression, temporally weighted regression, geographically-weighted regression, and GTWR models were used to construct factor regression models for land–sea convergenomics from four different perspectives (global, time, space, and space-time), using the previously mentioned independent and dependent variables. The estimations of these regression models were compared (S2 Table) to validate the superiority of the GTWR model, which was selected for this study.

### Temporal variations in factors affecting land–sea convergenomics

**Fig 5:**
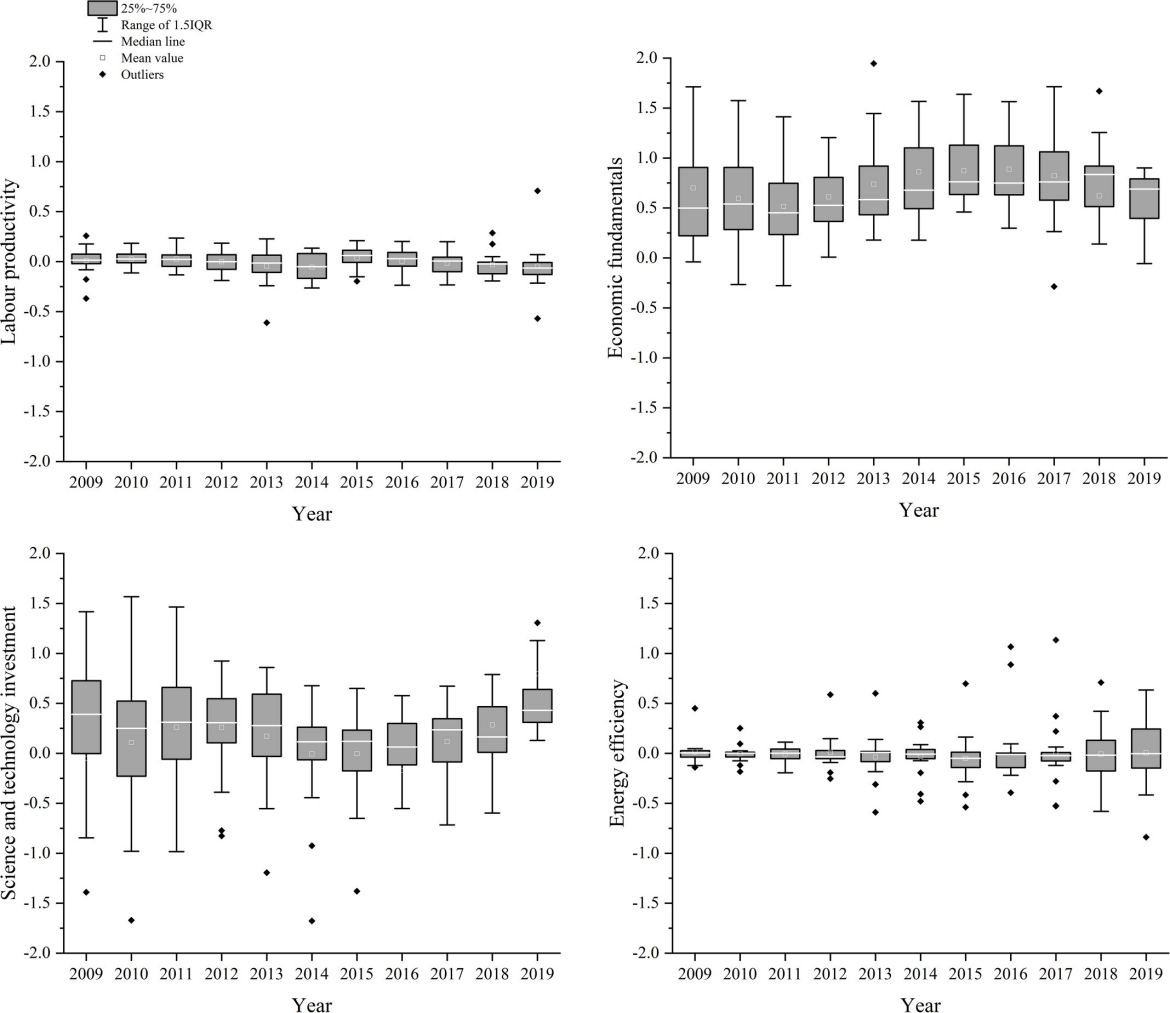
Boxing diagram of time variation in the land–sea convergenomics in Circum-Bohai Sea cities (2009–2019). (a) Labour productivity, (b) Economic fundamentals, (c) Science and technology investment, and (d) Energy efficiency.

Fig 5 shows a box plot of the spatiotemporal trends for the regression coefficients of the factors, showing that they significantly influenced the land–sea convergenomics in BER cities from 2009 to 2019. The regression coefficients were either positive or negative, implying positive or negative effects on land–sea convergenomics viscosity, depending on the region.

Labour productivity did not strongly affect viscosity, as its regression coefficient slowly but steadily became negative. Its dispersion increased from 2009 to 2014 and decreased after 2014, indicating that regional differences in labour productivity effects decreased over time. The effects of economic fundamentals on viscosity decreased slightly from 2009 to 2019; however, the mean value of its regression coefficient was consistently positive. The dispersion of the regression coefficient, which was initially large, also decreased during the latter half of the study period, indicating that economic fundamentals positively contributed to viscosity. Science and technology investment positively affected viscosity, as its regression coefficient remained positive despite fluctuations. Furthermore, the dispersion and range of its regression coefficient decreased and increased from 2009 to 2019, indicating that the effects of science and technology investment increased. Although energy efficiency only showed small fluctuations in the mean of its regression coefficient, it frequently switched between negative and positive values. Furthermore, the dispersion in its regression coefficient increased continuously throughout the study period, indicating that the effects of energy efficiency on viscosity were strongly differentiated in space and time, consistent with disparities in regional development, economic level, technical gaps, and energy use affecting regional disparities in land–sea convergenomics. Although this analysis revealed interesting trends, it was not possible to fully describe the characteristics of the factors using time series alone. Therefore, the spatiotemporal distribution of the degree of influence of the factors was investigated.

### Spatiotemporal differentiation of factors affecting land–sea convergenomics

**Fig 6:**
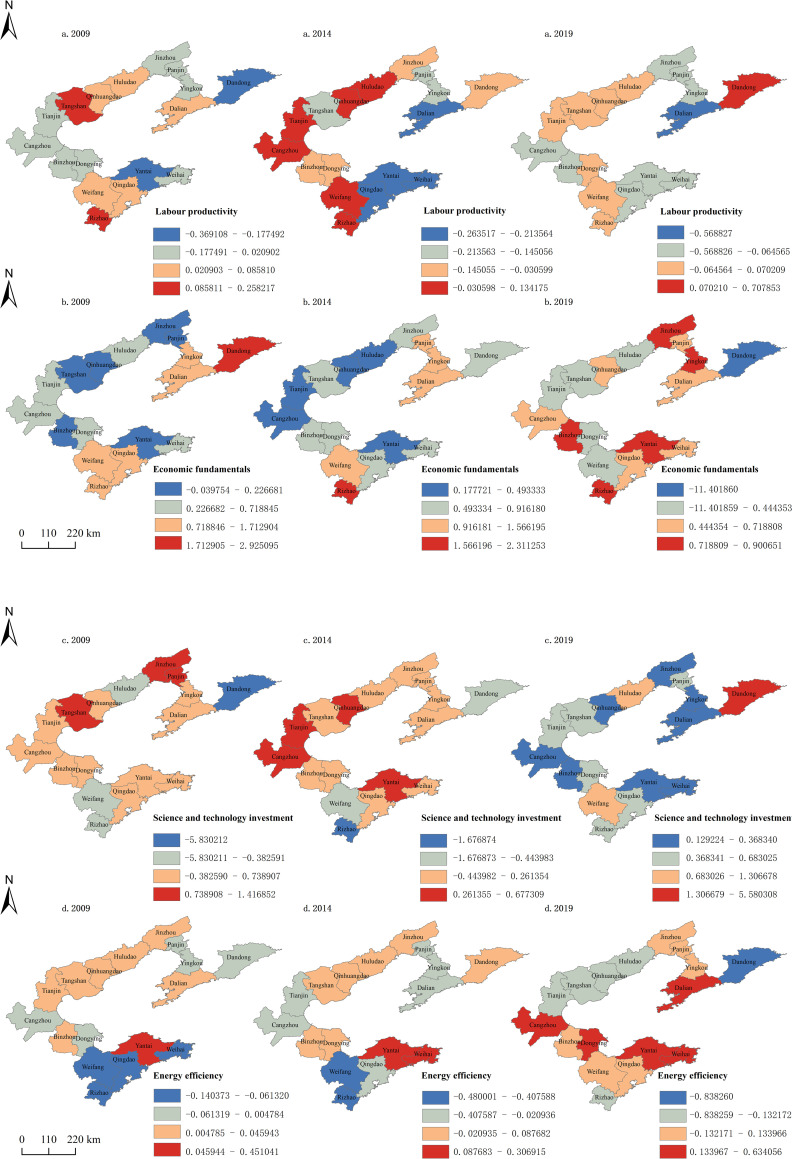
Distribution of regression coefficients for influencing factors of land–sea convergenomics GTWR in Circum-Bohai Sea cities. (a) Labour productivity, (b) economic fundamentals, (c) science and technology investment, and (d) energy efficiency.

The spatiotemporal distribution of GTWR-derived regression coefficients for the factors affecting land–sea convergenomics in BER cities is shown in Fig 6. The regression coefficient for labour productivity became negative, implying that improvements in labour productivity suppressed the development of land–sea convergenomics. The effects of labour productivity were positive in most cities in 2009; however, the number of cities with negative associations increased in 2014. Meanwhile, the coefficient in Tianjin became positive in 2014, possibly due to its high requirement for labour and the difficulty associated with changing old development models in large cities. By 2019, the correlation between labour productivity and viscosity was either negative or significantly weakened in most cities. Overall, the influence of labour productivity decreased in Dalian and Qingdao. Although there was a positive correlation between labour productivity and viscosity in Tianjin, the strength of the correlation was within a reasonable range.

Despite fluctuations in the regression coefficient for economic fundamentals, it was positively correlated with viscosity in most cities. In 2009, only Tangshan showed a weak negative correlation between economic fundamentals and viscosity. In 2014, the effects of economic fundamentals increased in most cities, except for Dandong and Tianjin, where the regression coefficient decreased significantly. Dandong, which has weak economic fundamentals, has focused on the development of these fundamentals instead of land–sea convergenomics, which led to a weak correlation. In cities that have strong economic fundamentals, such as Tianjin, further improvements in this aspect will have a limited effect on the development of land–sea convergenomics. In 2019, the effects of economic fundamentals were still predominantly positive, although weaker than earlier.

The regression coefficient for science and technology investment increased in a fluctuating manner, changing from negative to positive in most cities. In 2009, the regression coefficient was negative for Huludao, Dandong, Tianjin, Weifang, and Rizhao. In 2014, it became less negative in Huludao, Dandong, and Weifang, slightly negative in Dalian and Qingdao, and positive in Tianjin. In 2019, it became positive in all cities. Therefore, in all cities, science and technology investment was important for land–sea convergenomics. The cities in which its effects were strongest included Dandong, Huludao, and Weifang, which lie in the sphere of influence of the core cities. The influence of the core cities on science and technology investment grew steadily, however, remained within a reasonable range.

The regression coefficients for energy efficiency were highly variable, indicating that the effects of energy usage on land–sea convergenomics were complex. In 2009, the cities with the strongest negative correlations were Weifang and Rizhao (regression coefficients below -0.1), while Yantai showed the strongest positive correlation. In 2014, the regression coefficient became negative in Dalian, Panjin, and Jinzhou, and was largely negative in Tianjin. In 2019, the correlation changed from negative to positive in Qingdao, Dalian, and Cangzhou. Yantai maintained the strongest positive correlation and Dandong had the strongest negative correlation. Herein, energy efficiency was primarily evaluated based on coal. Ordinarily, improving energy efficiency should positively affect land–sea convergenomics; however, the development and resource requirements and dynamics of each city are highly variable. For example, Tianjin did not use coal on a large scale, as it shifted towards cleaner and more efficient energy sources. Nonetheless, the negativity of the correlation coefficient remained within a reasonable range. In cities with poorer development fundamentals, such as Dandong, the correlation was stronger as the reliance on coal was high.

In summary, the effects of each factor on land–sea convergenomics within the BER were differentiated in space and time, consistent with the spatiotemporal characteristics of their regression coefficients. The effects of labour productivity gradually weakened in the BER cities, while those of economic fundamentals on each city were still positive, although they weakened continuously during the study period. Furthermore, the weakening of its effects in core cities such as Tianjin, Dalian, and Qingdao spread to their surrounding cities. The effects of science and technology investment fluctuated but increased overall. In cities within the sphere of influence of the core cities, the correlation strengthened despite their limited level of development due to the influence of core cities. The effects of energy efficiency on each city were highly variable and complex, as the state of development and the resource dynamics of each city differ.

## Discussion

Our analysis indicated that BER cities have not attained optimal levels of land–sea convergenomics, as most cities have low viscosity scores with large disparities observed at the city level. Although few cities attained high viscosity levels and growth rates, it is not advisable to judge the development of a city based solely on its viscosity score and growth rate, as it is also necessary to consider the quality, structure, and vitality of its development. The development of land–sea convergenomics has been highly reliant on pre-existing economic resources and transportation networks, hindering new developmental models and innovations. Low and slow-growing resource development has been prevalent in many cities, indicating that more attention must be paid to the development of science and technology and the quality of the ecological environment.

The development of land–sea convergenomics in the BER was centred around the spheres of influence of three core cities: Tianjin, Qingdao, and Dalian. However, the influence of Dalian was notably weaker than that of Qingdao and Tianjin, as its development had stagnated. Therefore, more efforts should be dedicated to strengthening the influence of the core cities. The development of the core cities and their surrounding cities should be increased based on their circumstances to promote high-quality development of their marine and terrestrial economies.

GTWR analysis indicated that the effects of each factor on land–sea convergenomics in the BER were complex, as they were affected by regional disparities. The regression coefficients of the factors were spatiotemporally heterogeneous, as they could be either positive or negative, depending on the city and time. Herein, the concept of land–sea convergenomics was proposed based on detailed analyses of previous theoretical frameworks for developing marine and terrestrial economies. The connotations and mechanisms of this theoretical framework require further research. Using the systems development process as a guiding framework, we borrowed the concept of ‘viscosity’ from physics to construct a viscosity-based system of indices to evaluate land–sea convergenomics in the BER. This viscosity model was then combined with the space-time permutation scan statistic using SaTScan v9.5 software and the GTWR model to measure viscosity across the BER and analyse its influencing factors.

## Conclusions

Between 2009 and 2019, the land–sea convergenomics viscosity of the cities in the BER trended upward; however, significant differences were observed among them, as each city had different starting points and incremental changes. Although some cities showed large fluctuating uptrends, most were small with low viscosity (0.017–0.186).

During the 2009–2019 period, the viscosity variations of the BER cities showed some degree of spatiotemporal differentiation. The viscosity distribution was centred around three core cities, Tianjin, Qingdao, and Dalian.

The effects of labour productivity on viscosity weakened over time. Economic fundamentals positively affected viscosity in all cities, but the strength of its effect weakened over time. Although fluctuating, the effects of science and technology investment were strengthened, while its effect on viscosity in most cities changed from negative to positive. The effects of energy efficiency were highly variable and complex, possibly attributable to the cities having different states of development and resource dynamics.

The findings of this study indicated that land–sea convergenomics viscosity is a reliable and accurate method to theoretically evaluate and analyse land–sea convergenomics and could be used as a reference for future studies.

## Supporting information

S1 Fig Conceptual relationship of the land–sea convergenomics. (TIF)

S2 Fig Proportion of Bohai Rim city system-level index (2009–2019). (a) Development fundamentals, (b) Resource development, (c) Industrial linkages, and (d) Outcome creation.
(TIF)

S1 Table Level and type of land–sea convergenomics development in cities of Circum-Bohai Sea. (DOCX)

S2 Table Model test results. (DOCX)

S3 Table Spatiotemporal clustering results. (DOCX)

S1 Data (ZIP)
