# Supplementary figures and images for: Conceptualisation, estimation, and empirical analyses of land–sea convergenomics: A case study on Bohai Economic Rim cities
Source: PLoS One. 2022 Sep 20;17(9):e0274707. doi: 10.1371/journal.pone.0274707 (PMC9488836; doi:10.1371/journal.pone.0274707)

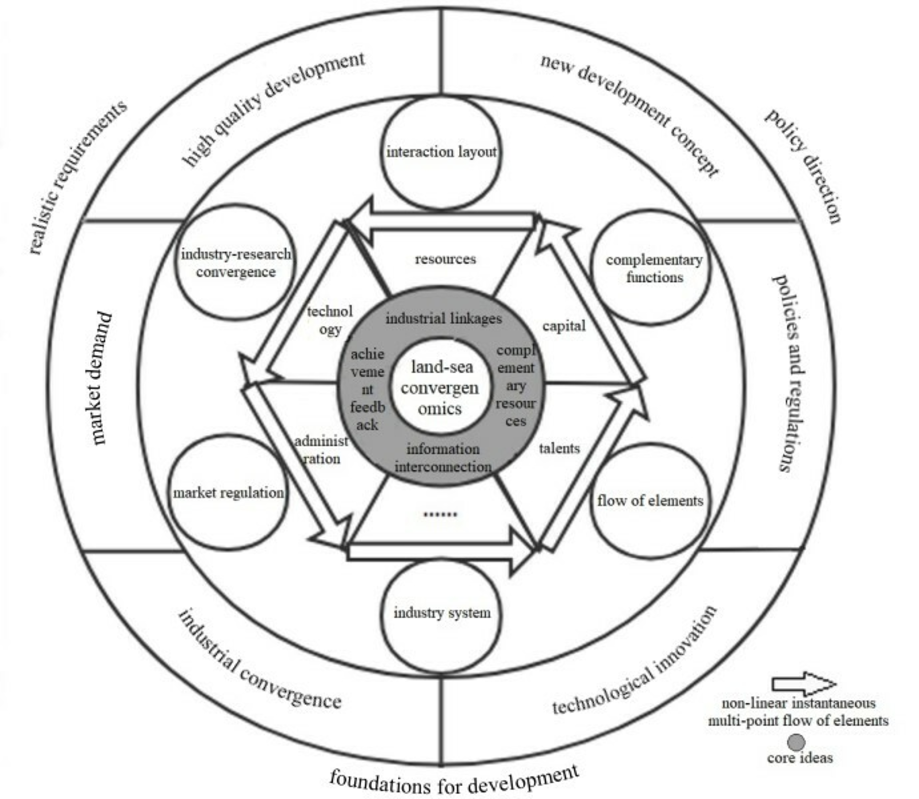

Supplement: S1 Fig — (TIF) [file pone.0274707.s001.tif]

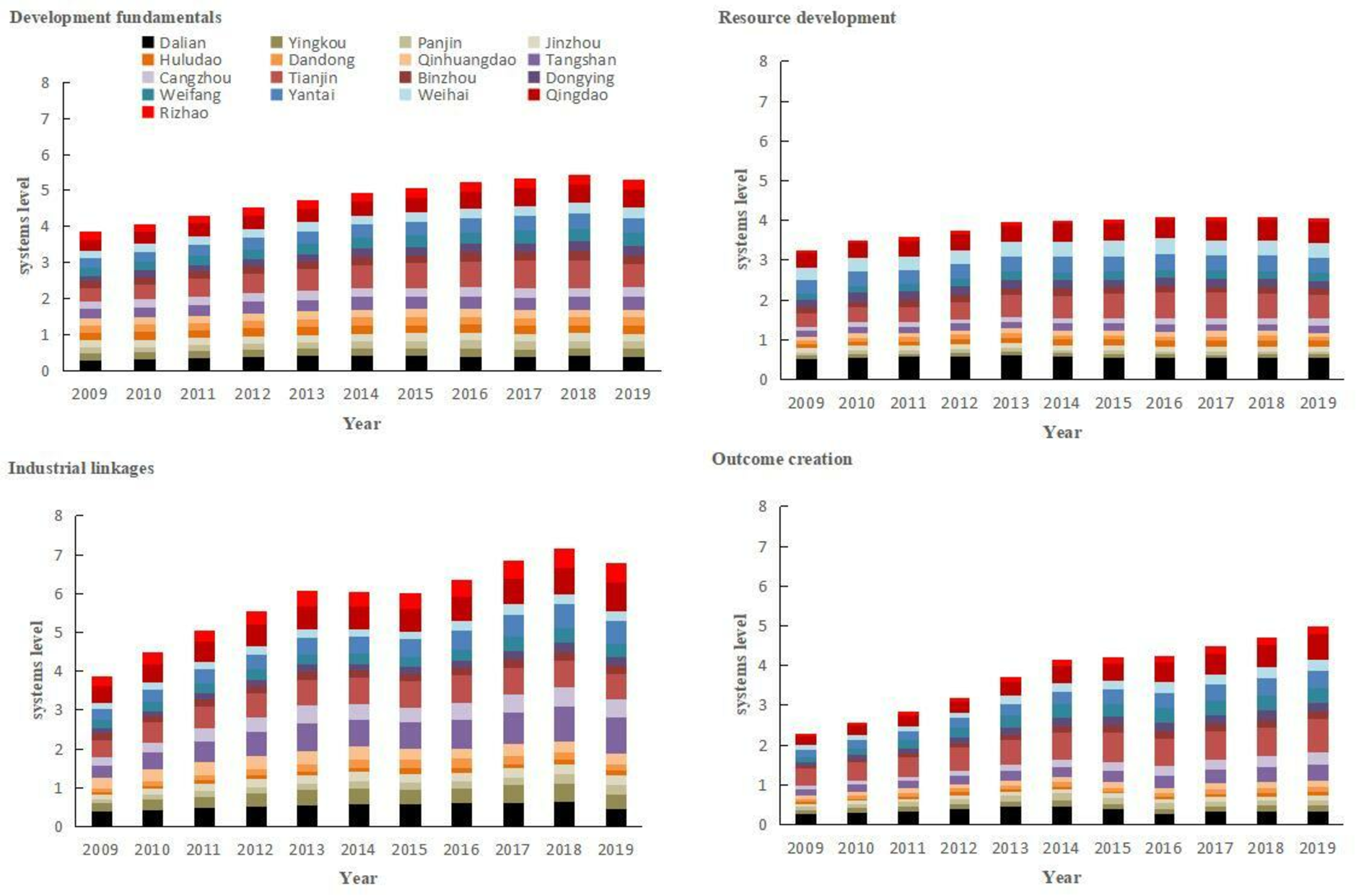

Supplement: S2 Fig — (a) Development fundamentals, (b) Resource development, (c) Industrial linkages, and (d) Outcome creation. (TIF) [file pone.0274707.s002.tif]
