# Supplementary material for: Conceptualisation, estimation, and empirical analyses of land–sea convergenomics: A case study on Bohai Economic Rim cities
Source: PLoS One. 2022 Sep 20;17(9):e0274707. doi: 10.1371/journal.pone.0274707 (PMC9488836; doi:10.1371/journal.pone.0274707)
Supplement: S1 Table — (DOCX) [file pone.0274707.s003.docx]

**Table S.1. Level and type of land–sea convergenomics development in cities of Circum-Bohai Sea.**

| Level | 0.70–1.00 | 0.50–0.70 | 0.30–0.50 | 0.10–0.30 | 0.05–0.10 | 0.00–0.05 |
| --- | --- | --- | --- | --- | --- | --- |
| Type | high | moderately high | moderate | moderately low | low | beginning |
