# Supplementary material for: Conceptualisation, estimation, and empirical analyses of land–sea convergenomics: A case study on Bohai Economic Rim cities
Source: PLoS One. 2022 Sep 20;17(9):e0274707. doi: 10.1371/journal.pone.0274707 (PMC9488836; doi:10.1371/journal.pone.0274707)
Supplement: S2 Table — (DOCX) [file pone.0274707.s004.docx]

**Table S.2. Model test results.**

| Models | OLS | TWR | GWR | GTWR |
| --- | --- | --- | --- | --- |
| R2 | 0.9643 | 0.9764 | 0.9930 | 0.9998 |
| AICc | -716.005 | -805.2983 | -1,075.2912 | -1,550.3682 |
