# Supplementary material for: Conceptualisation, estimation, and empirical analyses of land–sea convergenomics: A case study on Bohai Economic Rim cities
Source: PLoS One. 2022 Sep 20;17(9):e0274707. doi: 10.1371/journal.pone.0274707 (PMC9488836; doi:10.1371/journal.pone.0274707)
Supplement: S3 Table — (DOCX) [file pone.0274707.s005.docx]

**Table S.3. Spatiotemporal clustering results.**

| System | Timeframe | Coordinates/radius | Number of cases | Expected cases | P-value |
| --- | --- | --- | --- | --- | --- |
| Development fundamentals | 2009/1/1–2010/12/31  2017/1/1–2019/12/31 | 41.132588 N, 122.074086 E 246.38 km  36.715820 N, 119.167313 E 129.13 km | 23,256  33,682 | 20,727.87  31,095.31 | P<0.001  P<0.001 |
| Resource development | 2015/1/1–2019/12/31  2009/1/1–2013/12/31  2009/1/1–2012/12/31 | 39.113137 N, 117.208001 E 93.42 km  40.038564 N, 124.327755 E 294.91 km  36.715820 N, 119.167313 E 92.27 km | 38,674  48,122  15,790 | 34,533.54  44,610.45  14,972.19 | P<0.001  P<0.001  P<0.001 |
| Industrial linkages | 2018/1/1–2019/12/31 | 36.715820 N, 119.167313 E 271.16 km | 62,624 | 58,934.63 | P<0.001 |
| Outcome creation | 2009/1/1–2013/12/31  2016/1/1–2019/12/31 | 40.038564 N, 124.327755 E 304.68 km  36.715820 N, 119.167313 E 277.45 km | 40,851  98,663 | 33,903.04  91,015.54 | P<0.001  P<0.001 |
| Viscosity of land–sea convergenomics | 2009/1/1–2013/12/31  2017/1/1–2019/12/31 | 41.118676 N, 121.135824 E 294.91 km  36.715820 N, 119.167313 E 271.16 km | 27,524  46,068 | 22,646.01  42,118.76 | P<0.001  P<0.001 |
